# Comparative Study on Mechanical Properties of Sealing Grease Composed of Different Base Oils for Shield Tunnel

**DOI:** 10.3390/ma13030692

**Published:** 2020-02-04

**Authors:** Xiangqian Li, Yuyou Yang, Fan Li

**Affiliations:** School of Engineering and Technology, China University of Geosciences (Beijing), Beijing 100083, China; lxq@cugb.edu.cn (X.L.); fanli@cugb.edu.cn (F.L.)

**Keywords:** sealing grease, temperature sensitivity, cone penetration, tail shield, tunnel

## Abstract

This study proposes a novel sealing grease with improved mechanical properties and environmental performance. A series of sealing grease samples were made with different base oils, including mineral oil and renewable oil (vegetable oil and lard). In this study, thermogravimetric analysis (TGA) was conducted to study the adsorption capacity of the thickener to the base oil. The fluidity of the sealing grease was also tested at different temperatures. Furthermore, an exponential function was proposed for the flow rate of the sealing grease and the temperature. Moreover, a cone penetration test was conducted to study the consistency of the sealing grease. The results indicated that the capacity of the thickener to adsorb vegetable oil was greater than that of mineral oil, but less than that of lard. Additionally, the flow rate of the sealing grease increased with an increase in temperature. At a fixed temperature, the flow rate of the sealing grease increased with the base oil content. According to the exponential function, the composition of the base oil is the key factor that determines the temperature sensitivity of the sealing grease. In addition, the sealing grease made of vegetable oil has the minimum temperature sensitivity coefficient.

## 1. Introduction

Sealing grease is the only material for a shield tail seal that is vital for shielded tunnel boring machines (TBM). In recent years, the construction of tunnels has consumed a significant amount of sealing grease, owing to the widespread use of TBMs [1]. However, leakage often occurs at the shield tail, interfering with tunnel construction, and even causing accidents [2,3]. Moreover, the research on sealing grease is very scarce. Accordingly, this study introduces a type of sealing grease with good environmental performance and mechanical properties.

**Figure 1:**
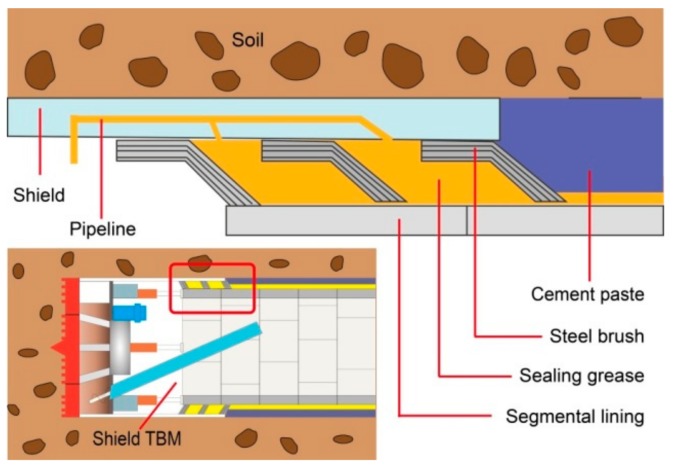
Schematic diagram of tail sealing for a shield TBM.

More specifically, during the construction of an underground tunnel, there will be an annular space between the shield tail of the shell and segment lining, as shown in Figure 1. At present, a brush seal has mainly been adopted for the annular space of TBMs [4,5]. Generally, there are three or four rounds of steel brushes installed in the annular space filled by the sealing grease. The sealing grease is a necessary material to seal the gap between the shield shell and the segment, and to prevent cement slurry or groundwater from entering the working space through the annular space. If the seal fails, the pressure of the backfill grouting will be insufficient, causing settlements at the ground surface and potentially even a collapse [6,7]. In addition, the sealing grease adhering to the outside of the concrete segments also has a sealing effect on the cracks between the concrete segments. The sealing grease and grouting material wrapped around the concrete segments remain underground permanently.

Sealing grease is a type of composite material that consists of base oil, thickener, viscosifier, filler, wood fiber, etc. [8,9,10,11]. The base oil and viscosifier play the role of matrix materials, because they are in continuous phase in the sealing grease. The filling materials play the role of reinforcement in composites, and are usually talc powder, calcium carbonate powder, bentonite, etc. Fiber is a commonly used reinforcing material in composite materials, and can improve the strength and durability of materials [12,13,14]. Adding wood fiber to sealing grease can improve its ductility and consistency.

The base oil of sealing grease is usually mineral oil. Its main component is polymer hydrocarbon. Mineral base oil is a type of non-renewable resource, and could cause pollution in production, processing, and application [15,16]. Some studies have tried to use degradable vegetable oil and its derivatives to replace mineral oil in preparing lubricating grease [17,18,19,20], but studies regarding using renewable oil in sealing grease have not been reported. Sealing grease is buried under the ground permanently after use. To avoid polluting the groundwater, it should be environmentally friendly. In this study, vegetable oil and lard were used as the base oil to make sealing grease, and can therefore improve the environmental performance of the sealing grease.

The mechanical properties of the sealing grease are vital for the sealing effect. However, there are few test methods to evaluate the mechanical properties of sealing grease. Accordingly, some test methods referenced tests from other materials that were similar to sealing grease, such as asphalt and lubricating grease. At room temperature, sealing grease is a type of sticky paste with good adhesion and extension. According to ASTM D2493, the Saal formula is recommended for the regression analysis of the temperature and asphalt viscosity. In addition, the temperature viscosity curve of asphalt can be obtained by the formula [21,22,23]. The temperature-viscosity characteristics have some impacts on the pumping performance [24]. As for sealing grease, a low temperature leads to high viscosity, and even pumping difficulties. In contrast, at a high temperature, sealing grease is soft and easy to pump, but has terrible sealing performance. Thus, a stable viscosity of the sealing grease is conducive to tunnel construction. Therefore, a melt flow rate meter was used to test the fluidity of sealing grease at different temperatures. Through a regression analysis, a correlation function of the sealing grease flow rate and temperature is presented. Furthermore, cone penetration is the other significant mechanical index of sealing grease. In the petrochemical standard, a cone penetration test can be directly used to evaluate the consistency of grease [25,26]. The cone penetration test also can be used to evaluate the shear property [27,28]. This study tested the cone penetration of sealing grease.

The main purpose of this article is to propose a novel sealing grease and investigate the effect of the renewable oil on the mechanical properties. Environmentally friendly sealing grease will be the development trend in the future as people pay more attention to environmental protection. Therefore, a series of sealing grease samples were made with the renewable oil. Through the thermogravimetric analysis, flow rate tests, and cone penetration tests, it was proven that the renewable oil can improve the mechanical properties of sealing grease in many aspects.

## 2. Materials and Methods

### 2.1. Materials

In this study, precipitated calcium carbonate (PCC), ground calcium carbonate (GCC), and bentonite were used as fillers. The particle size distribution of the fillers was measured by a laser particle size analyzer. Meanwhile, some physical parameters test results are listed in Table 1. 

Wood fiber is a reinforcement of seal grease, and can improve its mechanical properties. The average length of wood fiber used in the experiment was 3.02 mm. In addition, the ash content of the fiber was less than 5%. 

In this study, polyisobutylene and a base oil including mineral oil, vegetable oil, and lard were used as the matrix of the sealing grease. The mineral oil is a petrochemical product and is numbered 150 N. The main components of vegetable oil are soybean oil and rapeseed oil. Both vegetable oil and lard are edible. The viscosities of the vegetable oil and mineral oil used in the experiments are 32.8 mPa·s and 45.5 mPa·s, respectively, at a temperature of 40 °C. Polyisobutylene viscosity is 41.2 Pa·s at 40 °C. Calcium 12-hydroxystearate was used as a thickener to increase the consistency of the sealing grease, and was obtained by the saponification of calcium hydroxide and 12-hydroxystearic acid [29].

### 2.2. Mixture Design and Sample Preparation

#### 2.2.1. Mixture Design

To investigate the influence of the base oil on the sealing grease performance, different sealing grease samples with various base oils and contents were prepared. The components of base oil are shown in Table 2, in which renewable oil composed of vegetable oil and lard was numbered R, and mineral oil was numbered M0. According to the content of the base oil, the sealing grease samples were numbered as C1, C2, C3, and C4 corresponding to 15%, 17.5%, 20%, and 22.5% base oil, respectively, which includes the base oil adsorbed by the thickeners. For example, R0C3 means that the base oil content in the sample is 20%, and that the base oil is pure vegetable oil. Apart from the base oil, the relative proportion of the other ingredients remained constant, as shown in Table 3.

#### 2.2.2. Sample Preparation

**Figure 2:**
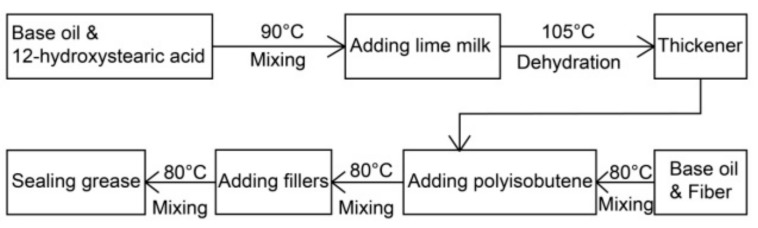
Schematic diagram of the manufacturing process of sealing grease.

**Figure 3:**
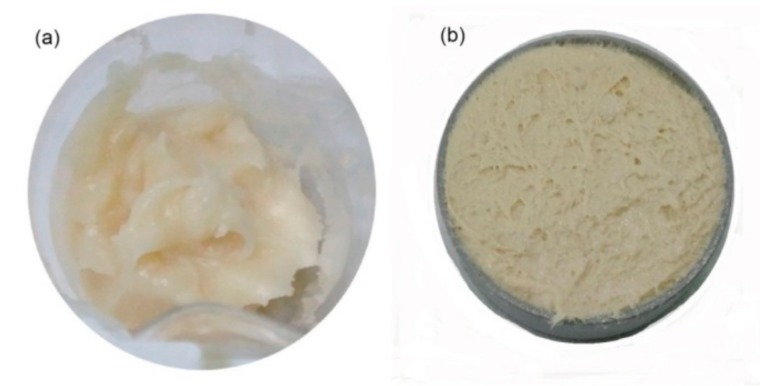
Samples preparation: (**a**) mixture of base oil and thickener; (**b**) sealing grease.

The manufacturing process of the sealing grease is shown in Figure 2. In the saponification, the mass ratio of calcium hydroxide (A.R.), 12-hydroxystearic acid, and base oil was 1:7:12, meaning an excess of calcium hydroxide. First, the base oil and 12-hydroxystearic acid were added to a beaker and heated up to 90 °C, at which time the 12-hydroxystearic acid had melted. Next, lime milk made of deionized water and calcium hydroxide with a concentration of 5 wt % was poured into the beaker, and stirred until the water became clear. After stirring, the water was poured out and the thickener was heated to 105 °C to remove the water until there were no more bubbles. Meanwhile, the wood fiber, base oil, and polyisobutylene were mixed in a beaker. The thickener was added to the beaker after the mixture was uniform. In the final steps, fillers were added to the mixture and mixed. The sealing grease sample was prepared at that time. If not specified, the temperature of each step above was 80 °C in a water bath. The images of the thickener and sealing grease are shown in Figure 3.

### 2.3. Test Methods

#### 2.3.1. Thermogravimetric Analysis and Microstructure

To analyze the adsorption capacity of the thickener to different base oils, the thickeners were tested by the Q500 thermogravimetric analyzer (TA Instruments, Battleboro, NC, USA) in nitrogen. The temperature increased from room temperature to 900 °C, with a heating rate of 10 °C/min. In addition, the microstructure of the thickeners was observed by a NeoScope JCM-5000 scanning electron microscope (Nikon Corporation, Tokyo, Japan).

#### 2.3.2. Fluidity Test

**Figure 4:**
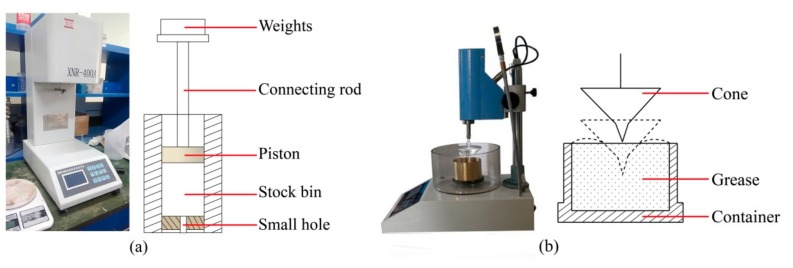
(**a**) Test method of melt flow rate meter; (**b**) Test method of cone penetration.

Fluidity is an important mechanical index of shield tail sealing grease, and can be reflected by a flow rate. Sealing grease is mainly pumped through pipelines to shield the tail. This means that sealing grease with poor fluidity is difficult to pump and clogs pipes easily, thereby causing construction difficulties [30]. However, excessive fluidity may reduce the sealing ability and increase the risk of leakage. The flow rate of the sealing grease in this study was tested by XNR-400A melt flow rate meter (Xiamen Jinheyuan Technology Co., Ltd., Xiamen, China) with a pressure of 1.0 MPa at different temperatures. The test method lets the sealing grease pass through a small hole with a length of 8 ± 0.025 mm and a diameter of 2.095 ± 0.005 mm under pressure as shown in Figure 4a. The mass of the sealing grease and time of extrusion were recorded to calculate the flow rate in units of g/min.

#### 2.3.3. Cone Penetration

Cone penetration is an index of consistency, and can indicate the rigidity of sealing grease. The smaller the cone penetration, the harder the sealing grease is. The thickener is the main factor that influences the cone penetration of the sealing grease. At present, the cone penetration of sealing grease in the market is between 200(1/10 mm) and 260(1/10 mm). A test of unworked penetration was conducted according to the standard ISO 2317 (2007) test methods, with a temperature of 25 ± 0.2 °C and a cone weighing 150 g. The schematic diagram of cone penetration test was shown in Figure 4b.

## 3. Results and Discussion

### 3.1. Thermal Gravimetric Analysis (TGA) Results of Thickener

**Figure 5:**
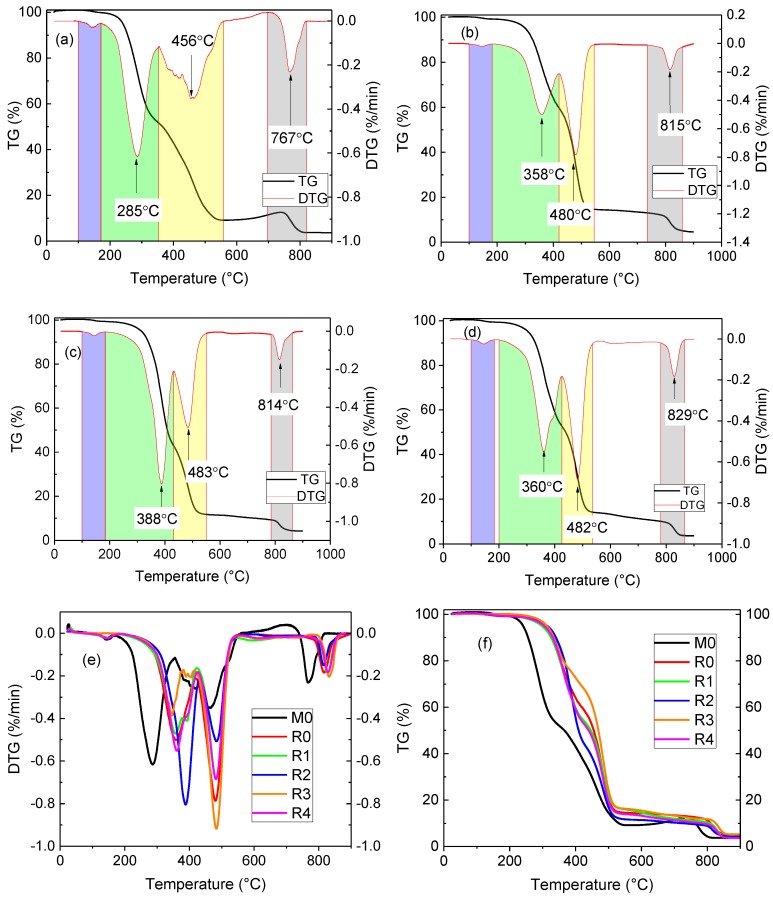
TGA results of thickener and base oil mixture: (**a**) M0; (**b**) R0; (**c**) R2; (**d**) R4; (**e**) DTG curves for all samples; (**f**) Thermogravimetry (TG) curves for all samples.

The thickener can absorb the base oil to increase the consistency of the sealing grease. The adsorption capacity of calcium 12-hydroxystearate to the base oil can be evaluated via thermogravimetric analysis. The test results are shown in Figure 5. Figure 5a is a thermogravimetric analysis curve of M0, which is the mixture of thickener and 150 N mineral oil. The thermal gravimetric analysis (TGA) curve of M0 can be divided into four phases. The first phase was from 130 °C to 200 °C, in which a small amount of water evaporated, and the loss counted for approximately 2% of the total mass. The second phase was from 200 °C, in which the base oil evidently volatilized and peaked at approximately 285 °C, and the weight loss was approximately 50%. The calcium 12-hydroxystearate decomposed in the third phase. The decomposition peaked at approximately 456 °C, and led to 40% weight loss. The fourth phase is mainly the thermal decomposition of the calcium carbonate, with a weight loss of approximately 6%. After the four phases, there is approximately 2% residue left.

The sample thickener R0 is made from vegetable oil and calcium 12-hydroxystearate. Figure 5b shows the thermogravimetric analysis curves of R0. It shows that the evaporation order of the constituents is similar to that of sample M0. First, a small amount of water evaporated before the heating to 200 °C. Then, the base oil evaporated. After that, the calcium 12-hydroxystearate decomposed because of heating, and decomposed completely at approximately 500 °C. At last, calcium carbonate decomposed completely at approximately 800 °C. The residue left is approximately 3% of the total mass, which is composed of calcium oxide. The difference is that the base oil of the sample R0 began to evaporate at 270 °C, and peaked at approximately 358 °C. As compared with sample M0, the initial temperature and peak temperature of evaporation exceed 70 °C and 73 °C, respectively. This showed that the thickener performed with a better adsorption capacity on vegetable oil than mineral oil.

Figure 5c shows the thermal analysis results of the sample R2. The largest evaporation rate of base oil occurred at approximately 388 °C, and was larger than that of the sample R0. Figure 5d shows the thermogravimetric analysis curve of sample R4, which was similar to that of the sample R0. However, the difference between R0 and R4 is that the differential thermogravimetric analysis (DTG) curve for R4 fluctuated at approximately 400 °C. The fluctuation may be caused by the different evaporation rates of vegetable oil and lard. The evaporation of lard did not form a single peak, meaning that the adsorption capacity of thickener on vegetable oil and lard was similar.

Figure 5e shows the DTG curves of all the thickener samples. It can be seen that the initial temperature and peak temperature of the evaporation of the base oil were the same from sample R0 to sample R4. The decomposition of calcium 12-hydroxystearate reached a peak at approximately 480 °C in all samples. The main reason for the different peak temperatures of the evaporation of the base oil is the different proportions of animal oil and vegetable oil used in the samples. Figure 5f shows the thermogravimetry (TG) curves of all of the thickener samples. It can be seen that the evaporation process of mineral oil occurs much earlier than those of vegetable oil and lard.

### 3.2. Microstructure of Thickeners

**Figure 6:**
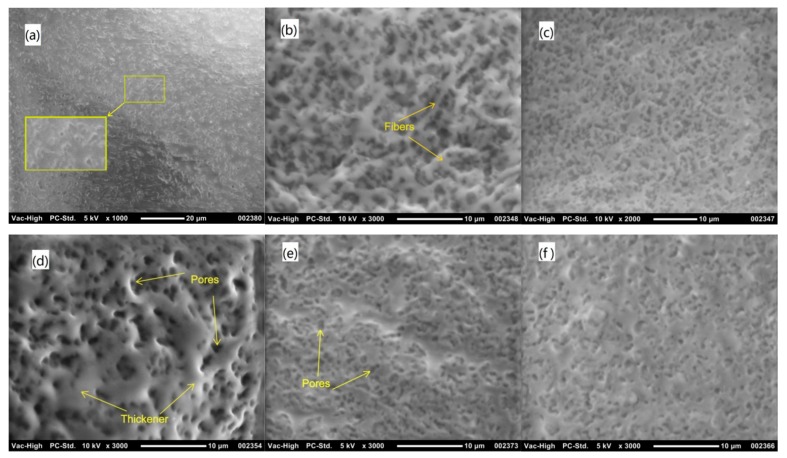
SEM images of thickener and base oil mixture: (**a**) M0; (**b**) R0; (**c**) R1; (**d**) R2; (**e**) R3; (**f**) R4.

The microstructure of thickener and base oil mixture were observed with a scanning electron microscope (SEM), as shown in Figure 6. All of the oils were adsorbed by calcium 12-hydroxystearate, and all of the thickeners were porous, as observed from the images. The microstructure of M0 was more compact than that of other thickeners. R0 was a three-dimensional network composed of fibers formed from calcium stearate. The fibers formed from the calcium stearate can still be observed in the microstructure of R1, which is similar to that of sponge. According to the SEM images, the microstructures of R2, R3, and R4 were honeycomb, and similar to that of M0. Furthermore, the number of pores successively reduced from R2 to R4. It can be observed from Figure 6e,f that the thickener was continuous, and the pores were independent.

Samples R1, R2, R3, and R4 contain lard and vegetable oil with different ratios, from 1/9 to 4/6. Therefore, it can be concluded that the thickener became more compact with an increase of lard content. As compared with the other thickeners, sample R0 has the loosest microstructure, because the thickener fibers entangled with each other and formed a three-dimensional network. The formation of fibers was caused by the cooling crystallization of calcium 12-hydroxystearate [31]. In addition, it has been reported that grease with a high entanglement structure displays a high yield stress in the transformation point when changing from a solid-like form to a liquid-like form [32].

### 3.3. Fluidity Test Results and Thermal Stability

#### 3.3.1. Regression Analysis

**Figure 7:**
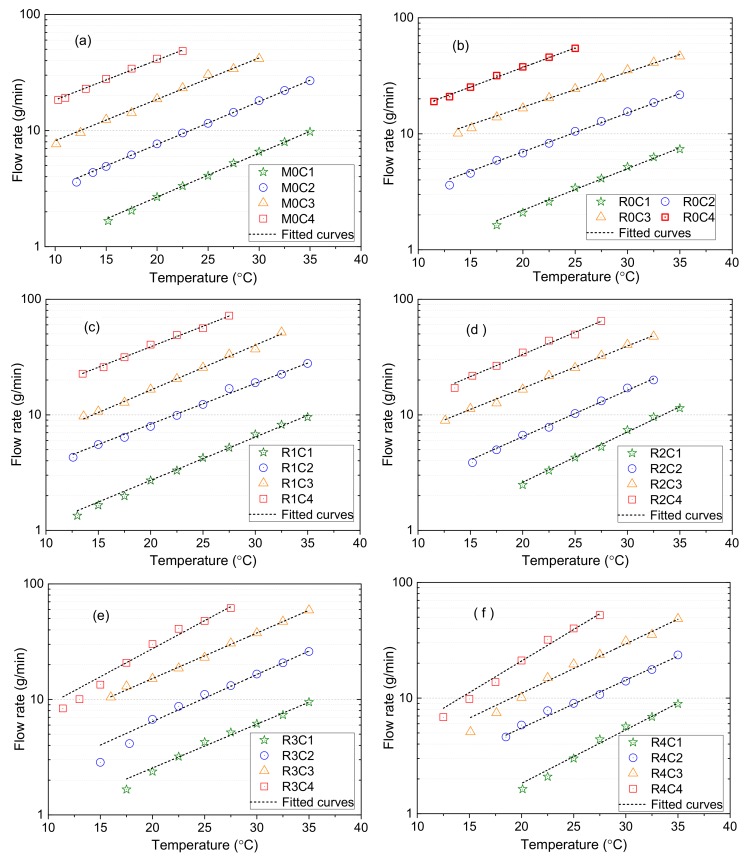
Fluidity test results for sealing grease with different temperatures: (**a**) M0; (**b**) R0; (**c**) R1; (**d**) R2; (**e**) R3; (**f**) R4.

The flow rates of the sealing grease samples were measured with the change of temperature, and the results are shown in Figure 7. The experimental results showed that the flow rate of the sealing grease increased with the increase of temperature, and has an exponential relationship with temperature. The exponential Equation (1) was used to fit the test results.

(1) $$f=e^{ax+b}$$

In Equation (1), *f* is the flow rate (g/min); *e* is the natural constant; *x* is the temperature (°C); and *a* and *b* are the regression coefficients.

The fitting results are shown in Figure 7 with dotted lines. The test results of sealing grease samples are well fitted to the function, and the correlation coefficient of M0C2 is 0.9994. The test results of samples R3 and R4 in the low temperature area deviate from the fitting curve. The fitting correlation coefficient of sample R3C4 is 0.9796, and is the smallest one. It can be seen from Figure 6 that the higher the base oil ratio in the sealing grease, the higher the flow rate will be. For example, with the sample R1 series sealing grease at the certain temperature of 25 °C, the flow rate of the sample R1C1 is 4.25 g/min. When the base oil content increased by 2.5%, the flow rate of R1C2 was 12.31 g/min, increasing by a multiple of 1.9. The results show that the content of base oil is the main factor affecting the fluidity of the seal grease. Without considering the influence of temperature, the regression coefficient *b* is the main parameter to determine the flow rate. Regression coefficient *a* indicates the sensitivity of the sealing grease flow rate to temperature. The greater the value of *a*, the greater the influence of temperature on the flow rate of the sealing grease. Considering that the environment for sealing grease is a normal temperature, the flow ability tests were carried out from 10 °C to 35 °C. When the temperature is above 35 °C, the fitting function may not be applicable.

#### 3.3.2. Effects of Base Oil on Fluidity

**Figure 8:**
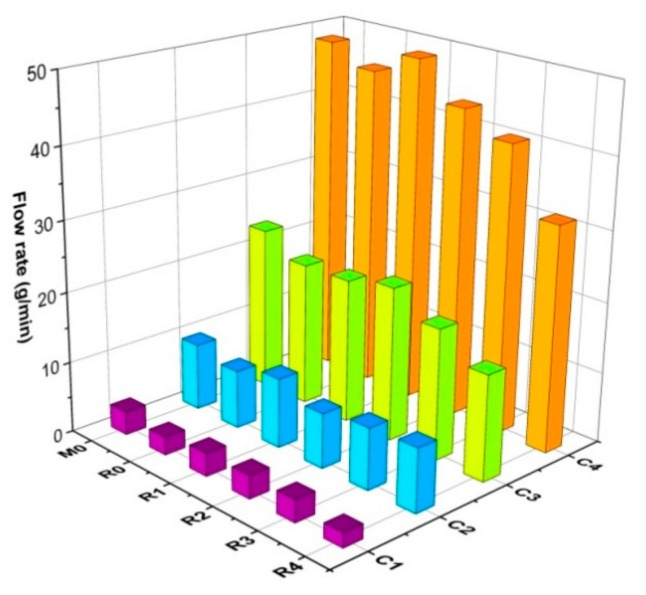
Flow rate of sealing grease at 22.5 °C.

To evaluate the impact of the content and type of the base oil on the fluidity of the sealing grease, the flow rate of all samples at 22.5 °C is shown in Figure 8. In the figure, it can be seen that the flow rate increased rapidly with the increase of base oil content. For example, when the base oil content increased from 15% to 22.5%, the flow rate of the R0 series samples increased from 2.6 g/min to 45.7 g/min, an increase by a multiple of approximately 18. Therefore, it can be concluded that the rheological properties of the sealing grease mainly depend on the content of the base oil.

When the base oil content was the same, in comparing the flow rate among the six series of sealing grease, R0 was always less than M0, which may be related to the viscosity of the base oil. It also can be observed that the flow rate of the sealing grease decreased with the increase of lard content. The reason for this phenomenon is that the viscosity of vegetable oil is higher than that of mineral oil, but is lower than that of lard. In contrast, the adsorption capacity of thickener to vegetable oil and the entanglement structure may be conducive to the displayed low flow rate for R0.

#### 3.3.3. Temperature Sensibility of Sealing Grease

**Figure 9:**
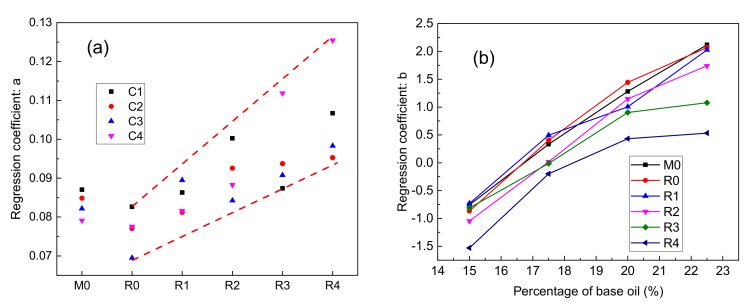
(**a**) Relationship between base oil and regression coefficient: *a*; (**b**) Effects of base oil content on regression coefficient: *b*.

As mentioned above, the regression coefficient *a* represents the sensitivity of the sealing grease flow rate to temperature. The greater the coefficient *a*, the more sensitive the flow rate to the temperature. Under the influence of weather, tunnel depth, ventilation, and other factors, the operating temperature of the sealing grease will change. Therefore, the lower the temperature sensitivity of the sealing grease, the better the safety of the shield tunnel construction. The relation between regression coefficient *a* and sealing grease type is shown in Figure 9a. It can be concluded that the temperature sensitivity of sealing grease is related to the type of base oil. The temperature sensitivity of M0 is higher than that of R0. This proves that the sealing grease prepared with vegetable oil has better temperature stability than that prepared with mineral oil. The regression coefficient *a* of R0 to R4 gradually increases, and the temperature stability gradually decreases. The percentage of lard in the base oils from R0 to R4 increased from zero to 40%. Therefore, the increase of temperature sensitivity coefficient may be related to the increase of the lard content in the base oil. Lard is solid at room temperature, and melts into liquid at approximately 30 °C. As a result, lard increases the sensitivity of sealing grease to temperature.

The test results of the flow rate indicated that the content of the base oil is the main factor influencing the flow rate. The relation between the regression coefficient *b* and the base oil content is shown in Figure 9b. The regression coefficient *b* increased with the increase of base oil content. The regression coefficient *b* of M0, R0, and R1 series grease is approximately linear with the percentage of base oil content. The base oil of M0 and R0 is vegetable oil and mineral oil that is liquid at room temperature. The base oil of R1 contains 90% vegetable oil and 10% lard. The increase of the base oil content leads to the increase of the low viscosity fluid composition of the sealing grease. Therefore, the regression coefficient *b* is approximately linear with the content of the base oil. The regression coefficients *b* of R2, R3, and R4 are non-linearly related to the percentage of base oil content. The base oils of the R2, R3, and R4 sealing greases contain 20%, 30%, and 40% lard, respectively. Lard is solid at room temperature, and thus may decrease the flow rate of the sealing grease. As the content of the base oil increases, the proportion of lard in the sealing grease also increases, so the regression coefficient *b* is non-linear with the content of the base oil.

### 3.4. Cone Penetration Test Results

**Figure 10:**
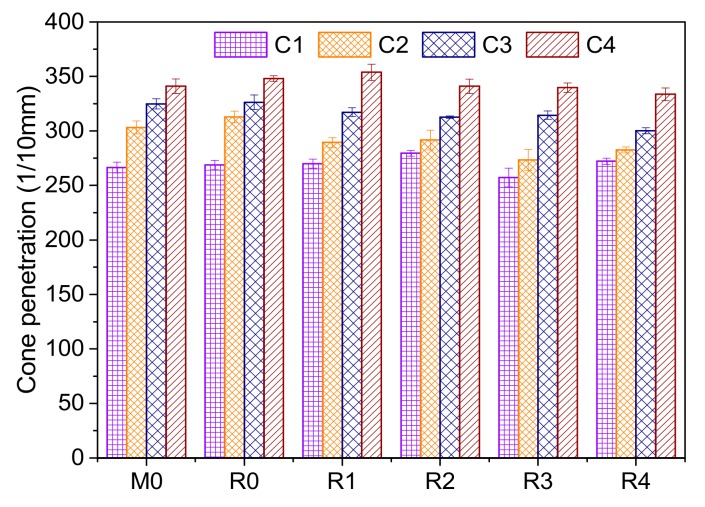
Cone penetration test results of sealing grease.

The cone penetrations of sealing grease were tested at 25 ± 0.2 °C. Each sample was tested three times. The average values and standard deviation of cone penetration were calculated as shown in Figure 10. The test results show that the cone penetration increases with the increase of base oil content. This indicates that the higher the base oil content, the softer the sealing grease will be. The statistical analysis of cone penetration shows that base oil content increased by 2.5%, and the sealing grease consistency increased by 2.46 mm on average. The cone penetration of M0 series sealing grease is close to that of R-series sealing grease. The liquid base oil among the fillers has a lubrication effect which can decrease the particle friction and shear stress. Therefore, increasing the base oil in sealing grease will lead to the increase of cone penetration. The cone penetration of sealing grease is generally 200(1/10 mm)–260(1/10 mm). If the cone penetration is too large, the grease pressure in the shield tail will be low and may lead to leakage.

## 4. Conclusions

To improve the environmental performance of shield tail sealing grease, mineral base oil, vegetable oil, and animal oil were used to make the sealing grease. The adsorption stability of the thickener to the base oil was studied by thermogravimetric analysis. The flow rate and cone penetration of the sealing grease were measured. The influence of temperature on the flow rate of the sealing grease was analyzed. The main conclusions of this research can be summarized as follows:

(1) Thermogravimetric analysis of the thickened material composed of base oil and thickener indicated that the weight loss process includes four stages: water evaporation, base oil evaporation, calcium 12-hydroxystearate thermal decomposition, and calcium carbonate decomposition. The peak temperature of vegetable oil evaporation is higher than that of mineral oil, indicating that the adsorption capacity of thickener on vegetable oil is stronger.
(2) SEM images show that the microstructure of the thickeners was different because of the base oil. The thickener composed of vegetable oil was a three-dimensional network. It became a honeycomb when the base oil contained lard and vegetable oil. Moreover, the microstructure of the thickener composed of mineral oil was more compact than other thickeners.
(3) The content of the base oil is the main factor affecting the fluidity of sealing grease. The higher the content of the base oil, the higher the flow rate of the sealing grease. The composition of the base oil is the factor that determines the temperature sensitivity of the sealing grease. The temperature sensitivity coefficient of sealing grease made from animal oil is the highest, whereas the temperature sensitivity coefficient of sealing grease made from vegetable oil is the lowest.
(4) The cone penetration of sealing grease increased with an increase of liquid base oil content. The liquid base oil distributed between the fillers has a lubrication effect, thus reducing the shear strength of the sealing grease. An increase in lard content could lead to a decrease in cone penetration.
(5) The study proved that the renewable oil can be used as a base oil to replace mineral oil. When the base oil content exceeded 17.5%, the consistency of sealing grease was too high and it may impair the sealing reliability. Furthermore, the suggested lard content in the base oil was less than 20%, because the lard increased the temperature sensitivity of sealing grease.

## Figures and Tables

**Table 1:** Physical parameters of fillers.

Parameters	PCC	GCC	Bentonite
Medium diameter (μm)	14.00	29.43	40.80
Mean diameter(μm)	23.02	39.80	47.90
Specific surface area (m^2^/g)	0.349	0.158	0.119

**Table 2:** Component of base oil.

Serial Number	R0	R1	R2	R3	R4	M0
Lard/Vegetable oil	0/10	1/9	2/8	3/7	4/6	mineral oil

**Table 3:** Ratio of other component in sealing grease.

Component	Thickener	Polyisobutene	Fiber	PCC	GCC	Bentonite
Relative percentage (%)	3.3	15.9	1.3	35.8	35.8	7.9

## References

[1] Gong C., Ding W., Soga K., Mosalam K.M., Tuo Y. (2018). Sealant behavior of gasketed segmental joints in shield tunnels: An experimental and numerical study. Tunn. Undergr. Space Technol..

[2] Zhang D., Huang Z., Yin Z., Ran L., Huang H. (2017). Predicting the grouting effect on leakage-induced tunnels and ground response in saturated soils. Tunn. Undergr. Space Technol..

[3] Yu C., Zhou A., Chen J., Arulrajah A., Horpibulsuk S., Horpibulsuk A.S. (2019). Analysis of a tunnel failure caused by leakage of the shield tail seal system. Undergr. Space.

[4] Edwards J.T. (1990). Civil Engineering for Underground Rail Transport.

[5] Jyo S. (1978). Brush-Type Packing Means for Shield Excavator. U.S. Patent.

[6] Jin D., Yuan D., Li X., Zheng H. (2018). Analysis of the settlement of an existing tunnel induced by shield tunneling underneath. Tunn. Undergr. Space Technol..

[7] Park H., Oh J.-Y., Kim D., Chang S. (2018). Monitoring and Analysis of Ground Settlement Induced by Tunnelling with Slurry Pressure-Balanced Tunnel Boring Machine. Adv. Civ. Eng..

[8] Wang S. (2016). Shield Machine Tail Sealing Grease Comprises Oil, Modifier, Filler, Fiber, Water-Absorbing Material Particles, and Additives. Patent.

[9] Xu H., Enemchukwu N., Zhong X., Zhang O., Fu Y. (2019). Deletion of M-opsin prevents “M cone” degeneration in a mouse model of Leber congenital amaurosis. Proceedings of the Primary Research on Preparation and Properties of Shield Tail Sealing Grease, 6th International Conference on Energy and Environmental Protection (ICEEP 2017).

[10] Ellenberger P., Egli H., Viscomi G. (2012). Sealing Paste. U.S. Patent.

[11] Franchini J., Merli L., Zangarini N., Bassi Li G. (2011). Tail Seals. U.S. Patent.

[12] Takaikaew T., Tepsriha P., Horpibulsuk S., Hoy M., Kaloush K.E., Arulrajah A. (2018). Performance of Fiber-Reinforced Asphalt Concretes with Various Asphalt Binders in Thailand. J. Mater. Civ. Eng..

[13] Wang H., Yang Z., Zhan S., Ding L., Jin K. (2018). Fatigue Performance and Model of Polyacrylonitrile Fiber Reinforced Asphalt Mixture. Appl. Sci..

[14] Wang Y., Guo P., Li X., Lin H., Liu Y., Yuan H. (2019). Behavior of Fiber-Reinforced and Lime-Stabilized Clayey Soil in Triaxial Tests. Appl. Sci..

[15] LU J., ZHANG Y., DUAN Q., LIU Y., YU K., WANG L., ZENG J. (2018). Structure Innovation and Commercialization Progress of Bio-based Lube Base Oil. Acta. Pet. Sin. (Pet. Process. Sect.).

[16] Bonal N.S., Paramkusam B.R., Basudhar P.K. (2018). Enhancement of surfactant efficacy during the cleanup of engine oil contaminated soil using salt and multi-walled carbon nanotubes. J. Hazard. Mater..

[17] Chang T.-S., Masood H., Yunus R., Rashid U., Choong T.S.Y., Biak D.R.A. (2012). Activity of Calcium Methoxide Catalyst for Synthesis of High Oleic Palm Oil Based Trimethylolpropane Triesters as Lubricant Base Stock. Ind. Eng. Chem. Res..

[18] Khemchandani B., Jaiswal A.K., Sayanna E., Forsyth M. (2014). Mixture of safflower oil and synthetic ester as a base stock for biodegradable lubricants. Lubr. Sci..

[19] Panchal T.M., Patel A., Chauhan D., Thomas M., Patel J.V. (2017). A methodological review on bio-lubricants from vegetable oil based resources. Renew. Sustain. Energy Rev..

[20] Del Mundo D.M.N., Sutheerawattananonda M. (2017). Influence of fat and oil type on the yield, physico-chemical properties, and microstructure of fat, oil, and grease (FOG) deposits. Water Res..

[21] Xu X., Yu J., Xue L., He B., Du W., Zhang H., Li Y. (2018). Effect of reactive rejuvenating system on physical properties and rheological characteristics of aged SBS modified bitumen. Constr. Build. Mater..

[22] Wang P.Y., Wen Y., Zhao K., Chong D., Wong A.S. (2014). Evolution and locational variation of asphalt binder aging in long-life hot-mix asphalt pavements. Constr. Build. Mater..

[23] Dong Z.-J., Zhou T., Wang H., Luan H. (2018). Performance Comparison between Different Sourced Bioasphalts and Asphalt Mixtures. J. Mater. Civ. Eng..

[24] Cong P., Luo W., Xu P., Zhao H. (2015). Investigation on recycling of SBS modified asphalt binders containing fresh asphalt and rejuvenating agents. Constr. Build. Mater..

[25] International Organization for Standardization (2007). Petroleum Products and Lubricants—Determination of Cone Penetration of Lubricating Greases and Petrolatum.

[26] Li B., Ren X., Li Y., Ma W., Li H. (2017). Evaluation and selection of sealants and fillers using principal component analysis for cracks in asphalt concrete pavements. J. Wuhan Univ. Technol. Sci. Ed..

[27] Qin X., Shen A., Guo Y., Li Z., Lv Z. (2018). Characterization of asphalt mastics reinforced with basalt fibers. Constr. Build. Mater..

[28] Lei Y., Wang H., Chen X., Yang X., You Z., Dong S., Gao J. (2018). Shear property, high-temperature rheological performance and low-temperature flexibility of asphalt mastics modified with bio-oil. Constr. Build. Mater..

[29] Saatchi A., Shiller P.J., Eghtesadi S.A., Liu T., Doll G.L. (2017). A fundamental study of oil release mechanism in soap and non-soap thickened greases. Tribol. Int..

[30] Song F., Wang Y., Wang J., Zhang J., Yang M. (2017). Test methods for grease of shield tail seal in shield engineering. Proceedings of the 6th International Conference on Energy and Environmental Protection.

[31] Shen T., Hu M., Liu R., Liu Q. (2011). The Influence of Static Thermal Degradation on Microstructure and Rheological Properties of Lithium-Calcium Base Grease. Tribology.

[32] Xu N., Wang X., Ma R., Li W., Zhang M. (2018). Insights into the rheological behaviors and tribological performances of lubricating grease: Entangled structure of a fiber thickener and functional groups of a base oil. New J. Chem..

